# Bentonite as eco-friendly natural mineral support for Pd/CoFe_2_O_4_ catalyst applied in toluene diamine synthesis

**DOI:** 10.1038/s41598-024-54792-5

**Published:** 2024-02-20

**Authors:** Alpár F. Hatvani-Nagy, Viktória Hajdu, Ágnes Mária Ilosvai, Gábor Muránszky, Emőke Sikora, Ferenc Kristály, Lajos Daróczi, Béla Viskolcz, Béla Fiser, László Vanyorek

**Affiliations:** 1 Higher Education and Industrial Cooperation Centre, University of Miskolc, Miskolc-Egyetemváros, 3515 Hungary; 2 Institute of Chemistry, University of Miskolc, Miskolc-Egyetemváros, 3515 Hungary; 3 Institute of Mineralogy and Geology, University of Miskolc, 3515 Miskolc-Egyetemváros, Hungary; 4 Department of Solid State Physics, University of Debrecen, P.O. Box 2, Debrecen, 4010 Hungary; 5 Ferenc Rakoczi II Transcarpathian Hungarian College of Higher Education, Beregszász, 90200 Ukraine; 6 Department of Physical Chemistry, Faculty of Chemistry, University of Lodz, 90-236 Lodz, Poland

**Keywords:** 2,4-DNT, Magnetic, Ferrite, Hydrogenation, Stability, Catalysis, Organic chemistry, Chemical synthesis

## Abstract

Toluene diamine (TDA) is a major raw material in the polyurethane industry and thus, its production is highly important. TDA is obtained through the catalytic hydrogenation of 2,4-dinitrotoluene (2,4-DNT). In this study a special hydrogenation catalyst has been developed by decomposition cobalt ferrite nanoparticles onto a natural clay-oxide nanocomposite (bentonite) surface using a microwave-assisted solvothermal method. The catalyst particles were examined by TEM and X-ray diffraction. The palladium immobilized on the bentonite crystal surface was identified using an XRD and HRTEM device. The obtained catalyst possesses the advantageous property of being easily separable due to its magnetizability on a natural mineral support largely available and obtained through low carbon- and energy footprint methods. The catalyst demonstrated outstanding performance with a 2,4-DNT conversion rate exceeding 99% along with high yields and selectivity towards 2,4-TDA and all of this achieved within a short reaction time. Furthermore, the developed catalyst exhibited excellent stability, attributed to the strong interaction between the catalytically active metal and its support. Even after four cycles of reuse, the catalytic activity remained unaffected and the Pd content of the catalyst did not change, which indicates that the palladium component remained firmly attached to the magnetic support's surface.

## Introduction

Toluene diamine (TDA)^1^ 1 plays an important role in the chemical industry being an essential intermediate in the production of polyurethane^2–4^. Worldwide the related business was valued at $1.8 billion in 2022, and significant quantities of TDA are used to produce dyes, hydraulic fluids, antioxidants, resins, and fungicides^5–8^. TDA is produced industrially using catalysts by hydrogenation of dinitrotoluene isomers^9^. The catalysts used are mainly transition metals or their compounds on various usually carbon-based supports^10–14^.

For efficiency the catalyst is reused several times, and it must be separated from the reaction medium at the end of the reaction. In continuous production, the catalyst lifetime determines the moment of separation. Removal is carried out in most cases by filtration. In conventional processes this leads to losses of catalysts. This can be avoided by using a suitable catalyst support and by preparing the catalyst particles in a suitable way.

A good option in terms of a carrier, but one that is little used in industrial practice, is the use of bentonite^15,16^. It is a noble clay with a high specific surface area due to its layered structure, making it an excellent catalyst support. The mineral includes tetrahedral (T) silicon oxide and octahedral (O) aluminum oxide structures which forms compact TOT layers, whose negative charge is compensated by Ca^2+^, Mg^2+^, and Na^+^ ions present in the interstitial space^17,18^. The layers are able to move apart in the presence of liquid, *i.e.* the mineral swells. The ions are mobile, which explains the high ion exchange capacity of bentonite. The mineral is well dispersible and has a high colloidal stability. Amines can also bind to the surface of bentonite, which increases the swelling of the mineral and its compatibility with the organic medium.

All the above beneficial properties led us to select bentonite as catalyst support during the solvothermal preparation of the catalyst^19,20^, which was used to produce highly magnetizable transition metal ferrites. In this way, filtration of the catalyst was eliminated, and process losses were significantly reduced. These ferrites show a certain reaction accelerating effect on their own, but the amine functionalized particles bind the palladium, and this significantly enhances the catalytic effect. Using this solvothermal method, we added the catalyst to the bentonite surface, multiplying the number of active centers and thus, increasing the lifetime and activity of the catalytic system.

## Materials and methods

Solvothermal synthesis of the amine-functionalized cobalt ferrite (CoFe_2_O_4_) nanoparticles was carried out starting from cobalt(II) nitrate hexahydrate (Co(NO_3_)_2_ · 6H_2_O, MW:291.03 g/mol, Sigma-Aldrich MO 63103, Saint Louis, USA) and iron(III) nitrate nonahydrate (Fe(NO_3_)_3_ · 9H_2_O, VWR International, Leuven, Belgium) precursors. As solvent and reactants ethylene glycol (HOCH_2_CH_2_OH, VWR Int. Ltd., F-94126 Fontenay-sous-Bois, France), ethanolamine (NH_2_CH_2_OH, Merck KGaA, D-64271 Darmstadt, Germany), and sodium acetate (CH_3_COONa, ThermoFisher GmbH, D-76870 Kandel, Germany) were used. Palladium(II) nitrate dihydrate (Pd(NO_3_)_2_·2H_2_O, Alfa Aesar LtD., MA 01835 Ward Hill, USA) was used to deposit Pd onto the ferrite catalyst supports. Nitrogen (purity 4.0, Messer) and hydrogen (purity 4.0, Messer) were used during the experiments. The catalytic tests were carried out by using 2,4-dinitrotoluene (DNT, C_7_H_6_N_2_O_4_, MW:182.13 g/mol), 2,4-diaminotoluene (TDA, C7H10N2, MW: 122.17 g/mol), 4-methyl-3-nitroaniline, 2-methyl-3-nitroaniline, and 2-methyl-5-nitroaniline (C_7_H_8_N_2_O_2_, MW:152.15 g/mol) from Sigma-Aldrich (Chemie Gmbh, D-89555 Steinheim, Germany). As an internal standard, nitrobenzene (C_6_H_5_NO_2_, MW:123.11 g/mol, Merck KGaA, D-64293 Darmstadt, Germany) was applied^21–27^.

The utilized bentonite originates from Transylvania, extracted from the surface mine of Muzsdaj III located in the Avas Mountain range (Romania—Bentoflux SA). The clay was allowed to de-tensioning for one month following extraction and was dried with microwaves to achieve a moisture content of 5% before utilization. Subsequently, it was stored in an exicator, underwent grinding in a laboratory grinding apparatus, and sieved through a 63 micrometric mesh. The resulting powder was used as a catalyst base. The applied process does not produce waste and the result is a natural ecological material. Basically, bentonite types are subdivided into two major groups. Wyoming type, and Cheto type, in function of Al^3+^ ion substitution with Mg^2+^ ions. In the Wyoming type the substitution is aleatory and rare. In the other case the Mg^2+^ ions have a defined position and orientation in the crystal and the substitution is more frequent. The used mineral is a Cheto type bentonite which is suitable for chemical reactions.

### Preparation of the Pd/CoFe_2_O_4_-bentonite catalyst

The deposition of cobalt ferrite nanoparticles onto the bentonite surface was carried out by a solvothermal method. Amine-functionalized CoFe_2_O_4_ spinel was synthesized by applying microwave (MW) irradiation-assisted solvothermal synthesis. In 75 ml ethylene glycol (EG) iron(III) nitrate nonahydrate (4.04 g), cobalt(II) nitrate hexahydrate (1.46 g), and sodium acetate (6.15 g) were dissolved. Additionally, 17.5 ml of ethanolamine was added to the solution of metal precursors. From the reaction mixture, 30–30 ml were measured in PTFE digestion tubes (volume: 100 ml). The reaction mixtures were placed in a CEM MDS 81 D microwave digestion instrument and treated at 200 °C (350 W) for 4 min under atmospheric pressure. After cooling bentonite (2.00 g) was added to the CoFe_2_O_4_-containing dispersion, which was dispersed by an ultrasound high energy homogenizer, Hielscher UIP100 Hdt. A tip homogenizer (1000 W, 20 kHz) with a Bs4d22 ultrasonic block sonotrode (D: 22 mm) was applied. The solid phase was separated from the dispersion media by centrifugation (4200 rpm for 10 min). Thereafter, cobalt ferrite-decorated bentonite was washed with distilled water several times, and the ferrite was easily separated with a magnet from the aqueous media. Finally, the ferrite was rinsed with absolute ethanol and dried overnight at 353 K. These ferrite-containing samples were used as magnetic catalyst support for the preparation of palladium-decorated spinel catalysts. The palladium nanoparticles were deposited onto the surface of the CoFe_2_O_4_-bentonite support by ultrasonication-assisted reduction from a methanolic solution of palladium(II) nitrate. The support (2.00 g) was dispersed in 200 mL of ethanol by ultrasonication with a Hielscher UIP1000 hDT homogenizer. For the dispersion of the ferrite-decorated bentonite, a 50 mol ethanolic solution of palladium(II) nitrate hydrate was added and it was treated by ultrasonication for 10 min. After magnetic separation of the catalysts, they were dried overnight at 353 K.

### Catalytic test: hydrogenation of 2,4-dintitrotoluene

The developed magnetic catalyst was tested in the synthesis of 2,4-toluenediamine (2,4-TDA) by hydrogenation of 2,4-dinitrotoluene (2,4-DNT). The hydrogenation tests were carried in 150 ml methanolic solution of 2,4-DNT (the concentration was 50 mmol/dm^3^) and 0.10 g catalyst was used in a Büchi Uster Picoclave reactor of 200 ml volume under constant agitation at 1000 rpm. The pressure of the hydrogenation was kept at 20 bar in all experiments and the reaction temperature was set to 213 K, 323 K, and 333 K, respectively. The sampling was carried out after 0, 5, 10, 15, 20, 30, 40, 60, 80, 120, 180, and 240 min during hydrogenation. As internal standard 5.0 µl nitrobenzene was added to 1.00 ml sample.

### Characterization techniques

The morphology and particle size were examined using high-resolution transmission electron microscopy (HRTEM) with a Talos F200X G2 electron microscope equipped with a field emission electron gun (X-FEG) and an accelerating voltage of 20–200 kV. The samples were prepared by applying a drop of aqueous suspension onto 300 mesh copper grids from Ted Pella Inc. X-ray diffraction (XRD) results were evaluated using Search/Match and Rietveld analysis to identify and quantitatively characterize the different phases present in the samples. A Bruker D8 Advance diffractometer with a Cu-Kα source operating at 40 kV and 40 mA in parallel beam geometry with a Göbel mirror and a Vantec detector was utilized. The average crystallite size of the domains was calculated by applying the mean column length calibrated method based on the full width at half maximum (FWHM) and the width of the Lorentzian component of the fitted profiles. The components of the raw bentonite were also determined by using a Bruker Vertex 70 FTIR spectrometer. The measurements were carried out in transmission mode, and in each case 10 mg sample was pelletized with 250 mg potassium bromide. The dispersibility and surface charge characteristics were examined. Electrokinetic (zeta) potentials were calculated based on electrophoretic mobility measurements applying laser Doppler electrophoresis by a Malvern Zetasizer Nano ZS equipment. The magnetic characterization of ferrite nanoparticles was carried out with self-developed (University of Debrecen) vibrating‐sample magnetometer system based on a water-cooled Weiss-type electromagnet. The powder samples were pelletized for the measurements with typical mass of 20 mg. The magnetization (M) was measured as a function of magnetic field (H) up to 10,000 Oe field strength at room temperature.

The amount of deposited palladium in the catalysts was analyzed using a Varian 720 ES inductively coupled optical emission spectrometer (ICP-OES). For the ICP-OES measurements, the samples were dissolved in aqua regia. Quantitative analysis of the samples after the hydrogenation tests was performed using an Agilent 7890A gas chromatograph coupled with an Agilent 5975C Mass Selective detector employing an RTX-624 column (60 m × 0.25 mm × 1.4 μm). The injected sample volume was 1 μL at a 200:1 split ratio, while the inlet temperature was set to 473 K. Helium was used as the carrier gas at a constant flow rate of 2.28 mL/min. The oven temperature was initially set to 323 K for 3 min and then heated to 523 K at a rate of 10 K/min, where it was maintained for another 3 min. The analytical standards for the main product, by-products, and intermediates were purchased from Sigma Aldrich and Dr. Ehrenstorfer Ltd.

## Results and discussion

### Characterization of the bentonit catalyst support

**Figure 1:**
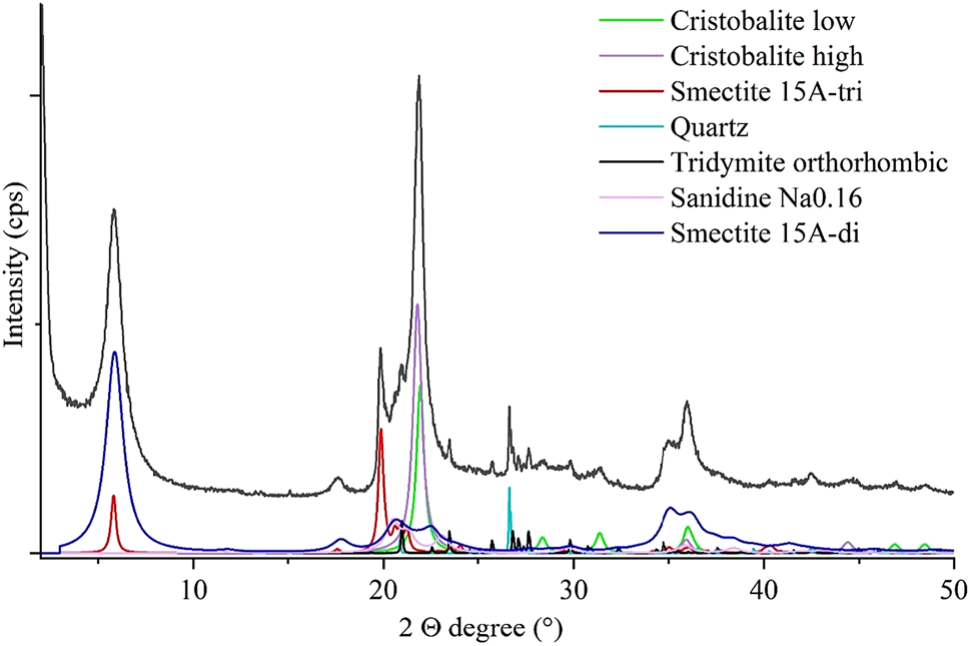
Rietveld refinement of the XRD pattern of the bentonite catalyst support.

The composition of bentonite applied as catalyst support by XRD is mainly montmorillonite (46.9 wt%) and cristobalite (25 wt%) with significant amorphous phase(s) (15.8 wt%), the remaining fraction being quartz (1.2 wt%), sanidine feldspar (2.4 wt%), and tridymite (8.7 wt%). The diffractogram deconvoluted by Rietveld refinement (Fig. 1) shows the presence of low and high temperature cristobalite, as well as di- and trioctahedral montmorillonite, however these details do not influence the ferrite deposition. The identified phases are supported by the FTIR results also, and the nature of amorphous phase(s) is revealed to be of hydrated aluminosilicate (clay) type, since no other compounds like carbonate or sulphate are observed. The average crystallite sizes of montmorillonite and cristobalite is < 50 nm with a median range ~ 20 nm. However, it is not possible to decide if these are single particle crystallite, on the contrary, they most likely form aggregates of several hundreds of nanometers. The presence of nanocrystalline cristobalite does not decrease the bentonite potential as catalyst support, since these SiO_2_ phases also might have surface OH groups and thus, increase adsorption capacity^28^.

**Figure 2:**
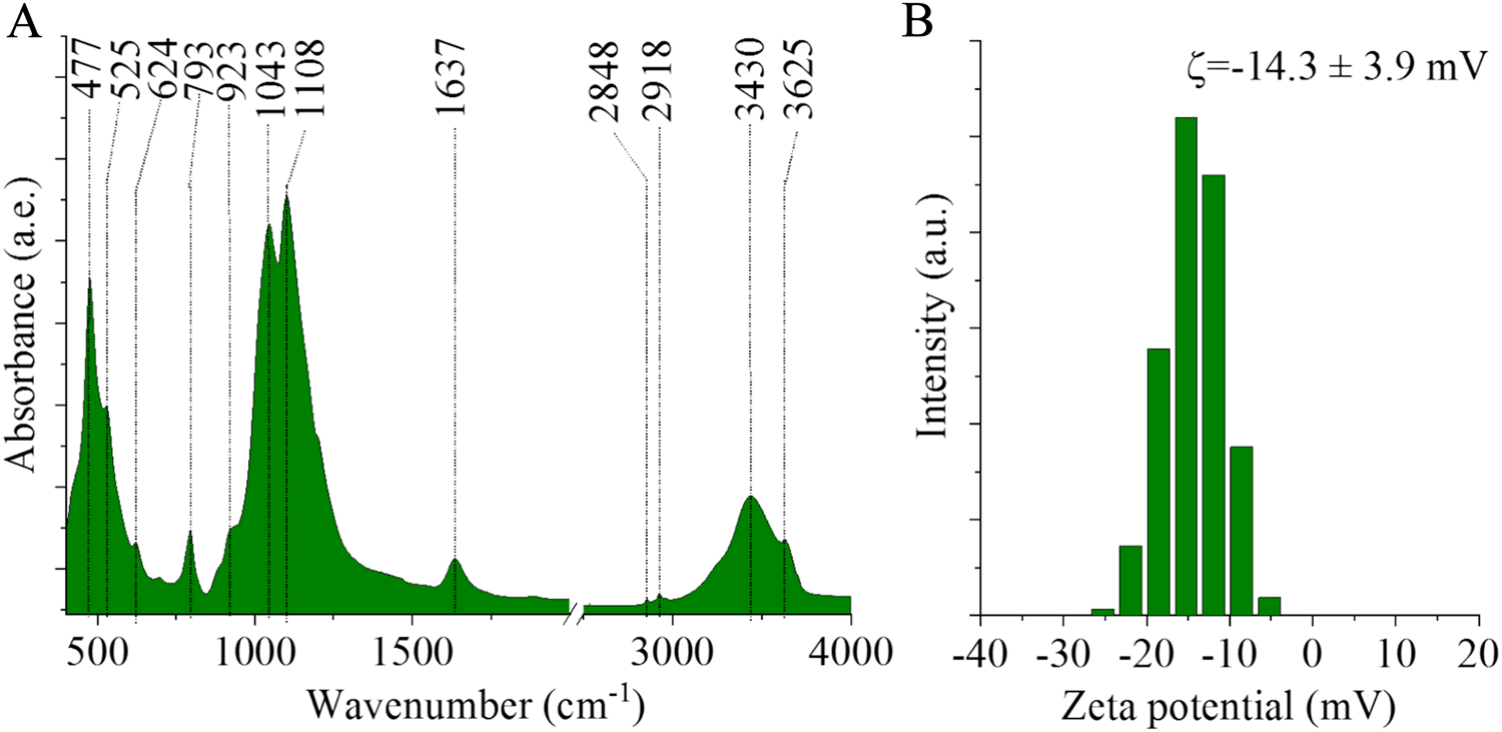
FTIR spectrum (**A**) and zeta potential distribution (**B**) of the bentonite sample.

Furthermore, FTIR measurements were used to identify the functional groups appearing on the bentonite sample, which basically defined the ion adsorption capacity and electrostatic interaction features of the clay with catalytically active metal ions (Fig. 2A). Bands at 477 cm^−1^, 525 cm^−1^, 624 cm^-1^, and 793 cm^−1^ originate from the Si–O–Al and Si–O–Si bending vibrations, respectively, representing the tetrahedral Si–O–Si groups and are mainly related to the cristobalite structure^29,30^. The presence of bands at 1108 cm^−1^, 1043 cm^−1^, and 923 cm^−1^ corresponds to the stretching vibrations of Si–O–Al, Si–O, Al–Al–OH, and Al–O, functional groups respectively, on tetrahedral and octahedral units^31^. The stretching of Si–O–Al of the clay structure results in an absorption band at 1108 cm^−1^. The bands at 3426 cm^−1^ and 1637 cm^−1^ are attributed to the vibrations of interlayer water molecules^32^, while the 3625 cm^−1^ band is corresponds to the octahedral layer’s O–H vibrations of Al–O–H or Si–O–H^33^. Two bands located at 2848 cm^−1^ and 2918 cm^−1^ are identified as the symmetric and asymmetric stretching vibrations of the –CH and –CH_2_ units, indicating the presence of organic compounds, probably cations from decaying organic matter. The presence of surface hydroxyl groups can be deprotonated, leading to the formation of negative charges on the clay particles, resulting in a negative zeta potential for these materials. To define the surface functional groups available for adsorption processes, the measurement of zeta potential is a suitable method. The average zeta potential was − 14.3 ± 3.9 mV for the bentonite samples, which indicates good wettability and dispersibility in water due to the polar nature of the clay surface (Fig. 2B). Dispersion stability and wettability are of fundamental importance during catalyst preparation, ensuring the homogeneous dispersibility of the catalytic metal ions (Pd^2+^) on the clay surface. Moreover, the ion exchange behavior of the surface functional groups supports the anchoring of metal ions.

### Characterization of the Pd/CoFe_2_O_4_-bentonite magnetic separable catalyst

**Figure 3:**
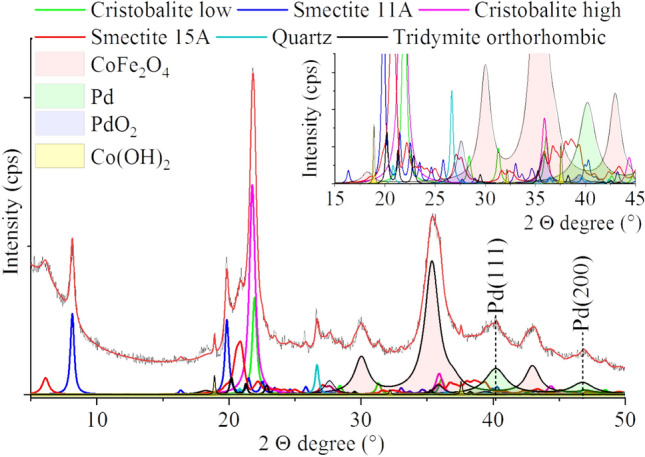
Rietveld refinement of the XRD pattern of the Pd/CoFe_2_O_4_-bentonite catalyst.

The phases of the catalyst were identified through XRD measurements. On the diffractogram, reflections of characteristic bentonite clays can be observed (Fig. 3) in different position and shape as compared to the original one (Fig. 1). Two types of smectite form are present as the results of Na-exchange from the solution, leading to smectite 11 Å and the remaining smectite 15 Å. The latter one has been delaminated by the sonication during catalyst preparation, as indicated by the broad and low basal peak. The increase of amorphous fraction from 15.8 to 26.2 wt% is due to the ultrafine delamination of montmorillonite, to lamellar thickness < 10 nm, thus behaving as X-ray amorphous material. The broadened diffuse diffraction rings related to the silicate phases support the presence of such material. The sharper peaks of the smectites at ~ 20° (2 Q) indicate anisotropic larger crystallites. The number of unit cells along the c-axis is about 4–5 approximately 60 nm size, while along the a- and b-axes, it is a few tens up to > 100 nm leading to thin and large platelets.

The (111) and (200) reflections of the elemental palladium are located at 40.2° and 46.1° 2 Q degrees in the case of the samples (PDF 46-1043). Based on the XRD measurement the average particle size of the Pd is 13 ± 2 nm. Palladium(IV) oxide was found also next to the elemental palladium according to the characteristic reflections identified at 27.5°(110), 35.4°(101) and 39.9° (200), respectively (PDF Card No. 04-002-4428). The average size of the palladium(IV)-oxid nanoparticles is 8 ± 2 nm.

The build-up of the cobalt ferrite nanoparticles was successful, as it can be seen on the diffractogram, peaks can be identified at 18.3°, 30.1°, 35.4° and, 43.0°, 53.3°, 56.8°, and 62.4° (2Q) which are characteristic for the (111), (200), (211) and, (220), (312), (303), and (224) reflections of the CoFe_2_O_4_ spinel phase (PDF Card No. 22-1086)^34,35^. In low quantity, cobalt(II) hydroxide phase was also detectable, and the corresponding reflections occurred at 18.9° (001), 32.2° (100) and, 37.6° (101), 50.9° (102), 57.3° (110), 60.9° (111), and 68.9° (103) 2 Q degrees (PDF Card No. 30-0443). The (111) and (200), and (220) reflections of the elemental palladium are located at 40.2° and, 46.1°, and 68.2° 2 Q degrees in the case of the samples (PDF 46-1043). Palladium(IV) oxide was found also next to the elemental palladium, according to the characteristic reflections identified at 27.5°(110), 35.4°(101), 39.9° (200), 54.3° (211), 57.0° (220), 61.1° (002), and 64.5° (310), respectively (PDF Card No. 04-002-4428).

**Figure 4:**
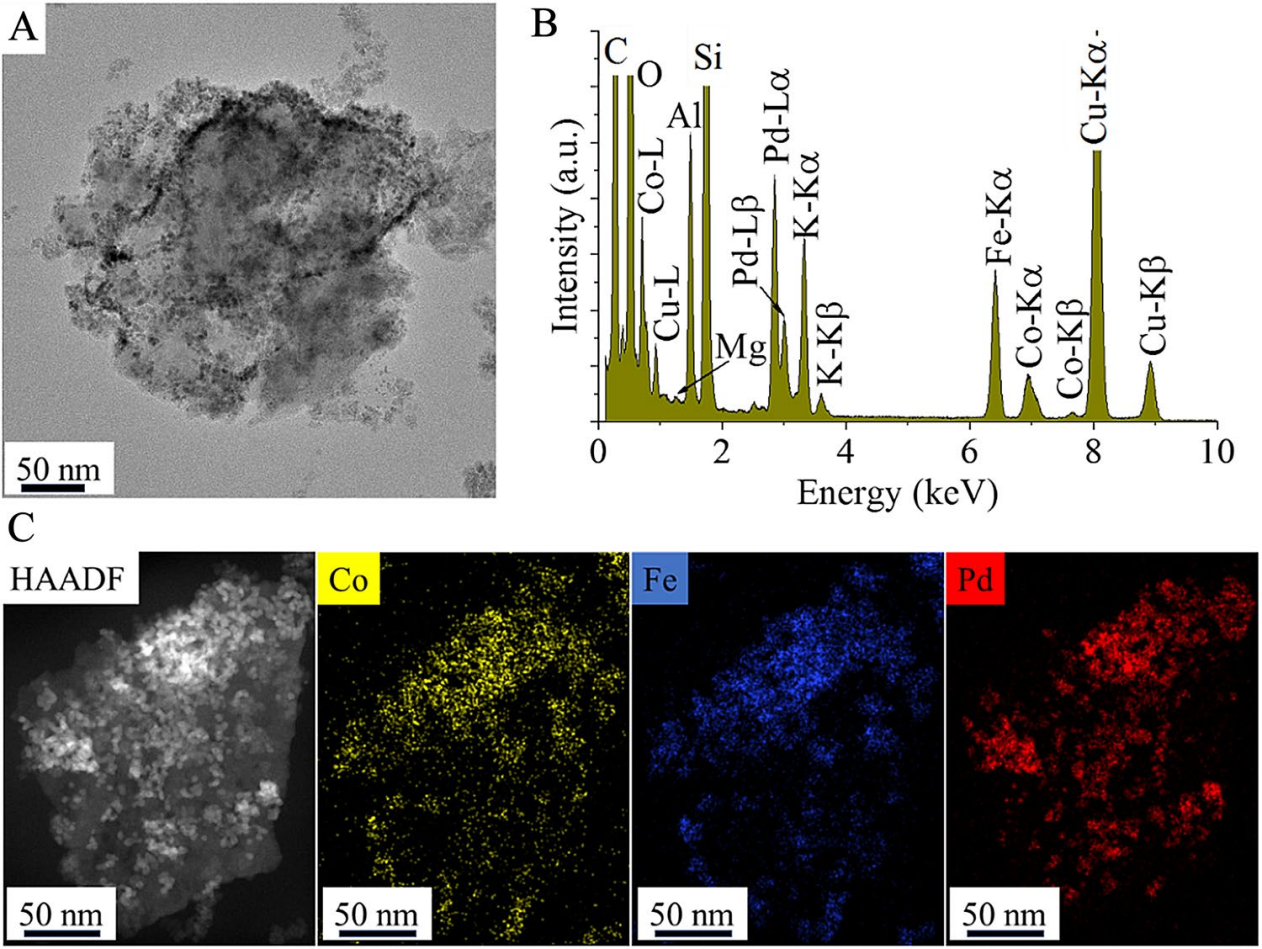
TEM image (**A**), EDS spectrum (**B**), and HAADF image with elemental maps (**C**) of the prepared Pd/CoFe_2_O_4_-bentonite catalyst.

On the HRTEM image, the palladium and cobalt ferrite nanoparticles are clearly observed and located on the surface of the clay layer (Fig. 4A). Moreover, the wide-thin plates of montmorillonite which formed by delamination as a result of the high-energy ultrasonic treatment during the deposition of palladium nanoparticles are also visible. This observation is consistent with the findings obtained via XRD measurements. In the EDS spectrum, all characteristic peaks of the elements composing the bentonite structure are identified, including Al, Si, K, and O (Fig. 4B). The presence of Fe and Co can be explained by the decomposition of CoFe_2_O_4_ nanoparticles onto the surface of the bentonite. The palladium phase, as a catalytically active metal, is also identified by EDS. The presence of the copper peak originates from the copper grid, which serves as the sample holder for the electron microscope. The intense carbon peak can be attributed to the adsorption of ethylene glycol and ethanolamine as reactants, which were used during the synthesis of the ferrite nanoparticles. The strong carbon signal from these adsorbed organic compounds is further confirmed by elemental mapping (Fig. 4C). Additionally, the elemental maps represent the distribution of cobalt and iron. It is noteworthy that Co is not only detected in areas where iron is present but also independently of iron on the clay mineral. This was confirmed by XRD measurements and cobalt is found in Co(OH)_2_ form. The HAADF image highlights particles with a stronger contrast which correspond to the palladium nanoparticles visible in the elemental map as well.

**Figure 5:**
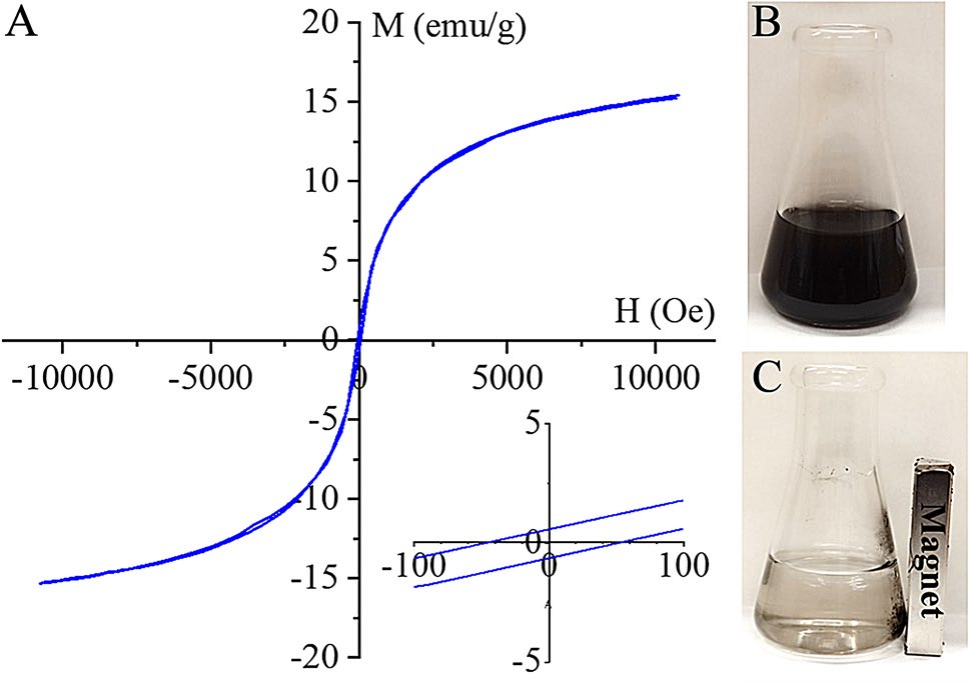
Vibrating-sample magnetometer (VSM) curve (**A**), excellent dispersibility (**B**) and magnetic separability (**C**) of the prepared Pd/CoFe_2_O_4_-bentonite catalyst sample.

Owing to the presence of –NH_2_ functional groups, the Pd(II)-ions efficiently anchored on the surface of the catalyst support, and thus, the Pd and PdO_2_ nanoparticles richly coated the support, which was confirmed by the element maps. For characterization of the magnetic behavior of the cobalt ferrite decorated bentonite, vibrating-sample magnetometer (VSM) measurement was carried out. On the magnetization curve, a narrow hysteresis loop with low coercivity (Hc: 52.1 Oe) and low remanent magnetization (Mr: 0.5 emu/g) was seen (Fig. 5). The saturation magnetization (Ms) was 15.4 emu/g of the magnetic catalyst support. The values of Hc and Mr were relatively small, which indicates the soft ferromagnetic nature of cobalt ferrite decorated clay mineral at room temperature. Narrow hysteresis loops indicate that the sample can be easily demagnetized.

### Catalytic tests of the developed Pd/CoFe_2_O_4_-bentonite magnetic separable catalyst

**Figure 6:**
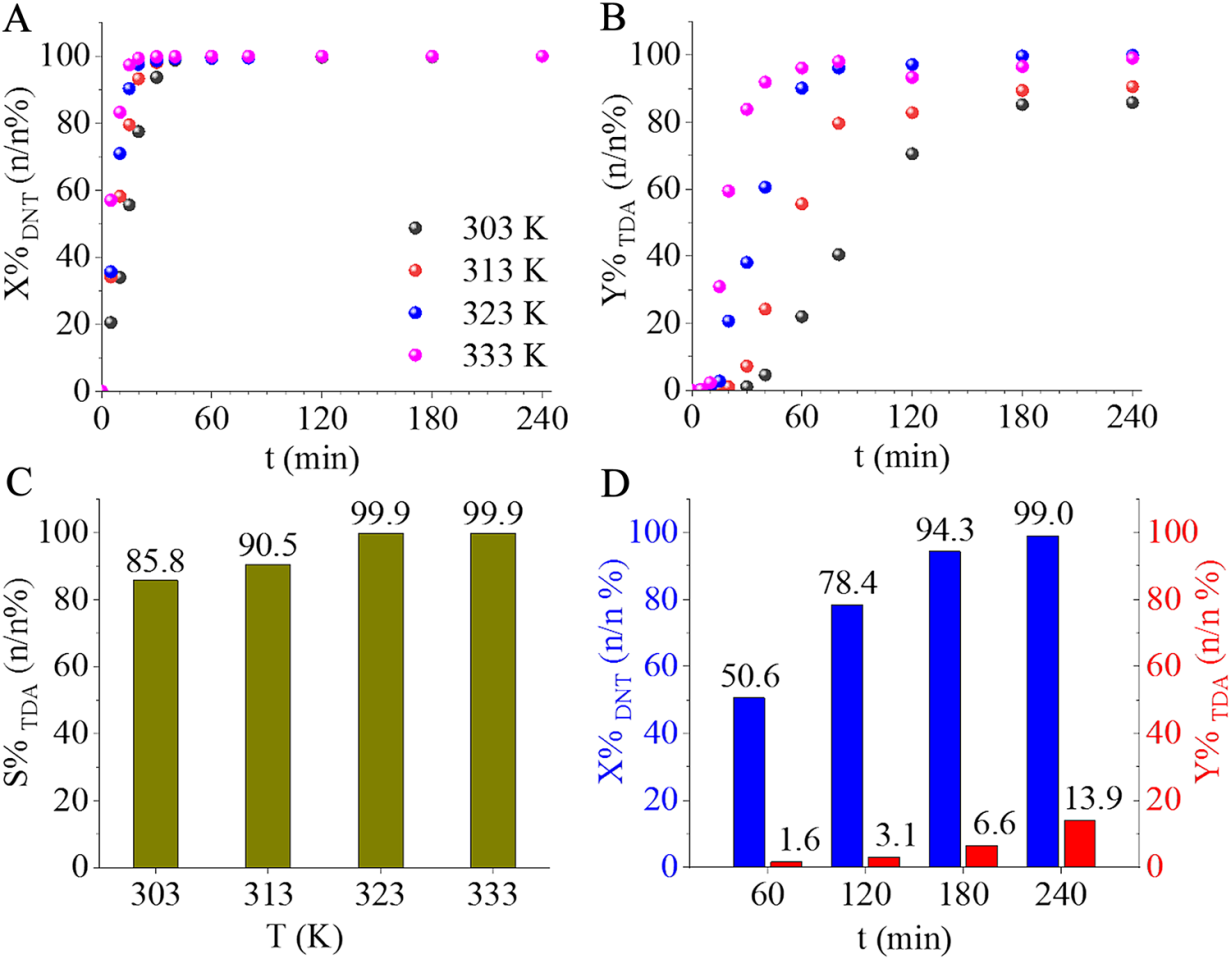
DNT conversion (**A**) and TDA yield (**B**) versus time of hydrogenation, and maximum TDA selectivity (S%) after 240 min, at 303 K, 313 K, 323 K, and 333 K (**C**). The catalytic activity of the palladium-free CoFe_2_O_4_-bentonite catalyst support was also compared to the developed catalyst at various temperatures (**D**).

The developed Pd/CoFe_2_O_4_-bentonite catalyst exhibited high catalytic activity at lower reaction temperature (303 K), as it is indicated by the 99 n/n % DNT conversion after 60 min of hydrogenation (Fig. 6A). Increasing the temperature did not significantly affect the conversion. The total amount of 2,4-DNT transformed into 3-methyl-4-nitroaniline (3M4NAN) and 2-methyl-5-nitroaniline (2M5NAN) as semi-hydrogenated intermediates, as well as 2,4-toluenediamine (2,4-TDA) in a short period. Regarding the product formation depending on hydrogenation time, the TDA yield varied at different reaction temperatures with higher temperature (333 K) leading to faster product formation (Fig. 6B). The selectivity of 2,4-TDA reached a maximum (> 99 n/n %) at 323 K and 333 K after four hours of hydrogenation (Fig. 6C). The elemental mapping and XRD results confirmed the presence of cobalt nanoparticles as potentially catalytically active phase. Consequently, the palladium-free CoFe_2_O_4_-bentonite magnetizable catalyst system was also tested for DNT (dinitrotoluene) hydrogenation at 20 bar H_2_ pressure and 333 K for 240 min (Fig. 6D). After 240 min of hydrogenation, the conversion of 2,4-DNT reached 99.0 n/n %, but the yield of 2,4-TDA (toluene diamine) was very low, only 13.9 n/n %. In this regard, the palladium-free support demonstrated high catalytic activity but unsatisfactory yield of the desired product, highlighting the need for a precious metal to enhance the activity. Overall, the Pd/CoFe_2_O_4_-bentonite catalyst exhibited remarkable catalytic behavior in 2,4-TDA synthesis.

**Figure 7:**
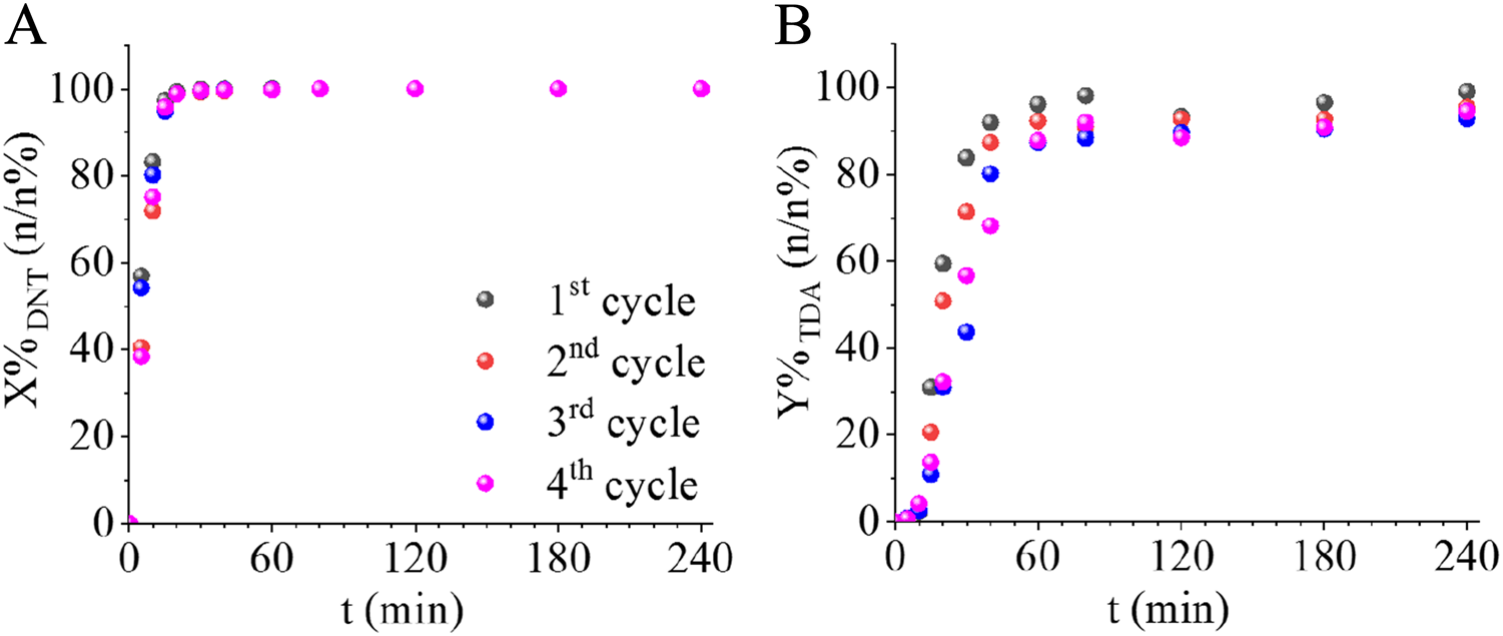
2,4-DNT conversion (**A**) and 2,4-TDA yield (**B**) versus time of hydrogenation up to 4 cycles at 333 K.

Long service life and reusability are key factors for catalysts. It is economically advantageous for the catalytically active phase to remain firmly attached to the catalyst support without leaching. Thus, strong interaction between the catalytic metals and their supports is highly advantageous and it can be achieved through the formation of functional groups capable of interacting with the catalytic phase. The amine group formed during the solvothermal synthesis on the surface of bentonite and the cobalt ferrite particles are well-suited for this purpose. To demonstrate this reuse tests were conducted subjecting the catalyst to 4 catalytic cycles at 333 K and under 20 bar of hydrogen pressure. The 2,4-DNT conversion remained consistent after four cycles (Fig. 7A).

Similarly, the 2,4-TDA yield did not show a significant decrease indicating that the catalyst retained its excellent catalytic activity even after multiple uses (Fig. 7B). No change was visible in DNT conversion, while minimal fluctuation (~ 4%) was measured in TDA yield after 240 min of hydrogenation (Table 1).

**Table 1:** DNT conversion and TDA yield values after 240 min of hydrogenation following each cycle.

	1st cycle	2nd cycle	3rd cycle	4th cycle
X_DNT_ (n/n%)	100	100	100	100
Y_TDA_ (n/n%)	99	95	93	95

It is important to emphasize that additional activation steps were not employed between the individual cycles and the catalyst was simply washed with methanol.

The palladium content of the magnetizable catalyst was measured before use and after four cycles by using the ICP-OES method to investigate possible palladium leaching. The palladium content of the fresh catalyst was 1.61 wt%, which did not show a significant decrease after multiple cycles (1.60 wt%). Based on the results of the reuse tests and ICP measurements, it can be stated that the developed catalyst is stable, owing to the strong interaction between the noble metal and its support^11–14,21,36^.

The developed Pd/CoFe_2_O_4_ bentonite-based catalyst was compared to 14 other systems^11,12,12,13,36^ applied in DNT hydrogenation by using the MIRA21 model^14^. It was found that the bentonite-based catalyst has a MIRA21 number of 11.09, which is just slightly lower than the best (11.50) and ranks as 8th within the list of 15 catalysts (see Supplementary Information).

## Conclusion

A special Pd/CoFe_2_O_4_-bentonite catalyst was developed by using bentonite, a clay mineral with a high specific surface area found in nature, as support. The support was decorated with cobalt ferrite and palladium nanoparticles. Even with the multiphase composition, such as silicates (montmorillonite and amorphous) and oxides (cristobalite and tridymite), precipitation and decoration were homogeneous. The successful processing can be attributed to the available oxygen containing groups on the surface, readily capturing metals and promoting in-situ ferrite crystallization.

The developed Pd/CoFe_2_O_4_-bentonite catalyst possesses excellent catalytic activity and can be easily separated by using magnetic field. The deposition of cobalt ferrite was achieved using a fast, microwave-assisted solvothermal method in an ethylene glycol phase, in the presence of ethanolamine during a 4-min reaction time. The presence of ethanolamine resulted in the formation of –NH_2_ functional groups on the ferrite surface, contributing to a strong interaction between bentonite and CoFe_2_O_4_ particles. The reduction of Pd(II) ions to elemental Pd nanoparticles on the cobalt ferrite-decorated bentonite surface was carried out using a sonochemical method in an ethanolic phase as the reductive medium.

The catalyst was tested in toluene diamine production and promoted complete conversion of 2,4-DNT, high yield, and selectivity towards 2,4-TDA. Furthermore, the catalyst demonstrated good performance during reuse tests, maintaining its catalytic activity even after the fourth cycle. No significant decrease in conversion or yield was observed when comparing unused and repeatedly used catalysts. It can be concluded that the palladium content remained stable. This indicates the successful production of a stable catalyst characterized by a strong interaction between the metal and its support. Furthermore, the Pd/CoFe_2_O_4_-bentonite catalyst was compared to 14 systems from the literature applied in DNT hydrogenation and it was revealed that its activity is close to other catalysts. All in all, the applied solvothermal process is suitable to produce stable catalysts with high specificity and long lifetime for industrial applications.

### Supplementary Information

Supplementary Information.

## Data Availability

The authors declare that the data supporting the findings of this study are available within the paper and its Supplementary Information files. Should any raw data files be needed in another format they are available from the corresponding author upon reasonable request.
