# Supplementary material for: Bentonite as eco-friendly natural mineral support for Pd/CoFe2O4 catalyst applied in toluene diamine synthesis
Source: Sci Rep. 2024 Feb 20;14:4193. doi: 10.1038/s41598-024-54792-5 (PMC10879086; doi:10.1038/s41598-024-54792-5)
Supplement: Supplementary file 1 — Supplementary Information. [file 41598_2024_54792_MOESM1_ESM.docx]

Bentonite as Eco-friendly Natural Mineral Support for Pd/CoFe_2_O_4_ Catalyst Applied in Toluene
Diamine Synthesis

Alpár F. Hatvani-Nagy^1^, Viktória Hajdu^1,2^, Ágnes Mária Ilosvai^1,2^, Gábor Muránszky^2^, Emőke Sikora^1,2^, Ferenc Kristály^3^, Lajos Daróczi^4^, Béla Viskolcz^1,2^, Béla Fiser^1,5,6,*^, László Vanyorek^2,*^

^1^Higher Education and Industrial Cooperation Centre, University of Miskolc, Miskolc-Egyetemváros, 3515, Hungary

^2^Institute of Chemistry, University of Miskolc, Miskolc-Egyetemváros, 3515, Hungary

^3^ Institute of Mineralogy and Geology, University of Miskolc, 3515 Miskolc-Egyetemváros, Hungary

^4^ Department of Solid State Physics, University of Debrecen, P. O. Box 2, Debrecen, 4010, Hungary

^5^ Ferenc Rakoczi II Transcarpathian Hungarian College of Higher Education, 90200 Beregszász, Ukraine

^6^ Department of Physical Chemistry, Faculty of Chemistry, University of Lodz, 90-236 Lodz, Poland

*****Correspondence: bela.fiser@uni-miskolc.hu & laszlo.vanyorek@uni-miskolc.hu

**Supplementary Information**

The MIRA21 model was developed in our group which can be applied to rank catalysts according to different parameters (<https://doi.org/10.3390/catal13020387>). We have compared 15 catalysts, including the currently developed bentonite-based one. It was found that the developed bentonite-based catalyst (indicated in blue in the table below) has an excellent MIRA21 number/score, 11.09, which is just slightly lower than the best (see **Tabel S1**).

**Table S1** Comparison of catalysts applied in DNT hydrogenation. The developed Pd/CoFe_2_O_4_ bentonite-based catalyst is indicated in blue.

| **CATALYSTS** | | | | | | |  |  |
| --- | --- | --- | --- | --- | --- | --- | --- | --- |
| **No.** | **CATALYST ID** | **Catalyst Name** | **Catalyst Support** | **Main Active Component** | **Known Par.** | **MIRA21 Number** | **Ref** |  |
| 1 | HDNT/MIS/2021/2/2 | Pt/CrO2 | Chromium(IV)-dioxide | Platinum | 15 | 11.50 | <https://doi.org/10.3390/ijms22115945> |  |
| 2 | HDNT/MIS/2021/2/1 | Pd/CrO2 | Chromium(IV)-dioxide | Palladium | 15 | 11.49 | <https://doi.org/10.3390/ijms22115945> |  |
| 3 | HDNT/MIS/2021/3/1 | Pd/NiFe2O4 | Nickel ferrite | Palladium | 15 | 11.45 | <https://doi.org/10.1016/j.mtchem.2021.100470> |  |
| 4 | HDNT/TIA/2020/1/3 | 15Pt/ZrO2-300 | Zirconium-dioxide | Platinum | 13 | 11.44 | <https://doi.org/10.1002/jctb.6359> |  |
| 5 | HDNT/TIA/2020/1/4 | 15Pt/ZrO2-400 | Zirconium-dioxide | Platinum | 13 | 11.43 | <https://doi.org/10.1002/jctb.6359> |  |
| 6 | HDNT/TIA/2020/1/2 | 15Pt/ZrO2-200 | Zirconium-dioxide | Platinum | 13 | 11.42 | <https://doi.org/10.1002/jctb.6359> |  |
| 7 | HDNT/MIS/2021/1/2 | Pd/maghemite | Maghemite | Palladium | 15 | 11.35 | <https://doi.org/10.1016/j.catcom.2021.106342> |  |
| 8 | HDNT/MIS/2024/4/1 | Pd/CoFe2O4-bentonite | CoFe2O4-bentonite | Palladium | 14 | 11.09 |  |  |
| 9 | HDNT/TIA/2020/1/5 | 45Pt/ZrO2-300 | Zirconium-dioxide | Platinum | 13 | 11.06 | <https://doi.org/10.1002/jctb.6359> |  |
| 10 | HDNT/TIA/2020/1/6 | 60Pt/ZrO2-300 | Zirconium-dioxide | Platinum | 13 | 11.01 | <https://doi.org/10.1002/jctb.6359> |  |
| 11 | HDNT/TIA/2020/1/7 | 85Pt/ZrO2-300 | Zirconium-dioxide | Platinum | 13 | 11.00 | <https://doi.org/10.1002/jctb.6359> |  |
| 12 | HDNT/MIS/2021/3/2 | Pd/CoFe2O4 | Cobalt ferrite | Palladium | 15 | 10.84 | <https://doi.org/10.1016/j.mtchem.2021.100470> |  |
| 13 | HDNT/SHA/2012/1/1 | Ni/HY catalyst | HY molecular sieve | Nickel | 15 | 10.77 | <https://doi.org/10.4028/www.scientific.net/AMR.512-515.2381> |  |
| 14 | HDNT/MIS/2021/1/1 | Pt/maghemite | Maghemite | Platinum | 15 | 10.67 | <https://doi.org/10.1016/j.catcom.2021.106342> |  |
| 15 | HDNT/MIS/2021/3/3 | Pd/CuFe2O4 | Copper ferrite | Palladium | 15 | 10.48 | <https://doi.org/10.1016/j.mtchem.2021.100470> |  |
